# Behavioral Sciences in the Optimization of Pharmacological and Non-Pharmacological Therapy for Type 2 Diabetes

**DOI:** 10.3390/bs11110153

**Published:** 2021-11-03

**Authors:** António Lopes, Fátima Roque, Sandra Morgado, Cristina Dinis, Maria Teresa Herdeiro, Manuel Morgado

**Affiliations:** 1 Pharmaceutical Services of Unity Local of Health of Guarda (ULS da Guarda), 6300-035 Guarda, Portugal; antonio.clopes@ulsguarda.min-saude.pt (A.L.); cristina.dinis@ulsguarda.min-saude.pt (C.D.); 2 Health Sciences Faculty, University of Beira Interior (FCS-UBI), 6200-506 Covilhã, Portugal; mmorgado@ipg.pt; 3 Research Unit for Inland Development, Polytechnic Institute of Guarda (UDI-IPG), 6300-559 Guarda, Portugal; 4 Health Sciences Research Centre, University of Beira Interior (CICS-UBI), 6200-506 Covilhã, Portugal; 5 Pharmaceutical Services of University Hospital Center of Cova da Beira, 6200-251 Covilhã, Portugal; sandracristinamorgado@gmail.com; 6 Institute of Biomedicine, Department of Medical Sciences (iBiMED-UA), University of Aveiro, 3810-193 Aveiro, Portugal; teresaherdeiro@ua.pt

**Keywords:** type 2 diabetes, behavioral sciences, lifestyle, self-control, antidiabetic therapy

## Abstract

Type 2 diabetes mellitus is one of the main chronic diseases worldwide, with a significant impact on public health. Behavioral changes are an important step in disease prevention and management, so the way in which individuals adapt their lifestyle to new circumstances will undoubtedly be a predictor of the success of the treatments instituted, contributing to a reduction in the morbidity and mortality that may be associated with them. It is essential to prepare and educate all diabetic patients on the importance of changing behavioral patterns in relation to the disease, with health professionals assuming an extremely important role in this area, both from a pharmacological and non-pharmacological point of view, and also ensuring the monitoring of the progress of these measures. Diabetes is a chronic disease that requires a high self-management capacity on the part of patients in order to achieve success in treating the disease, and non-adherence to therapy or non-compliance with the previously defined plan, together with an erratic lifestyle, will contribute to failure in controlling the disease. The lower adherence to pharmacological and non-pharmacological treatment in diabetes is mainly correlated to socio-economic aspects, lower health literacy, the side effects associated with the use of antidiabetic therapy or even the concomitant use of several drugs. This article consists of a narrative review that aims to synthesize the findings published in the literature, retrieved by searching databases, manuals, previously published scientific articles and official texts, following the methodology of the Scale for Assessment of Narrative Review Articles (SANRA). We aim to address the importance of behavioral sciences in the treatment of diabetes, in order to assess behavior factors and barriers for behavior changes that have an impact on the therapeutic and non-therapeutic optimization in patients with type 2 diabetes mellitus control.

## 1. Introduction

The high incidence of chronic diseases and non-adherence (or inadequate adherence) to pharmacological and non-pharmacological treatments by patients represent a serious problem in terms of public health, constituting a growing challenge for the different institutions and health professionals involved.

Failure to comply with the defined therapeutic plans can result in clinical complications, physical and emotional stress caused by the need for successive hospitalizations, consequently leading to economic and financial implications at the level of health systems [1,2].

Diabetes is one of the chronic diseases with the greatest impact worldwide in terms of morbidity and mortality, being closely associated with poor eating habits, sedentary lifestyle, smoking, alcoholism, increased life expectancy and the genetic component of each individual [3,4,5]. It has a multifactorial nature, is complex and heterogeneous, whose pathophysiology is closely associated with the development of insulin resistance and hyperglycemia, contributing to the impairment of the normal functioning of different organs and physiological systems, with type 2 diabetes mellitus (DM2) being the most prevalent form (about 90% versus 10% of type 1) [6,7].

**Figure 1:**
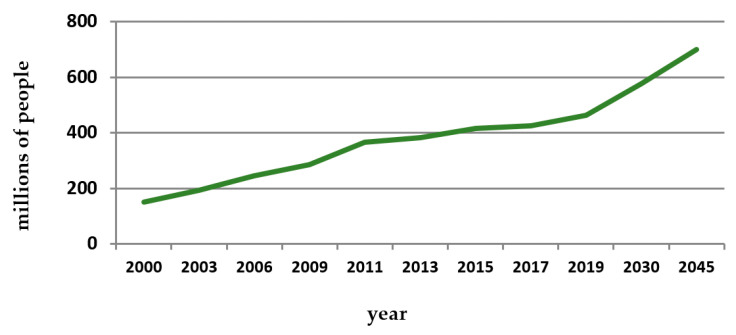
Estimated number of adults with diabetes (in millions) since 2000 to 2045. Source: IDF Diabetes Atlas editions [8].

According to the International Diabetes Federation (IDF), for 2019, both forms of diabetes caused 4.2 million deaths, and there are around 463 million adults aged between 20 and 79 years diagnosed with the disease, a number that will likely increase to 700 million by 2045 (Figure 1) [8,9]. Furthermore, the disease is likely to be under-represented, as there are suggestive data indicating that 1 in 3 patients are under-diagnosed [8].

Sarwar N. et al. (2010) demonstrated through a meta-analysis that there is a strong association between DM2 and an increased risk of coronary heart disease, ischemic stroke, retinopathy, nephropathy, foot ulcers (diabetic foot) and lower limb amputations [10,11].

**Figure 2:**
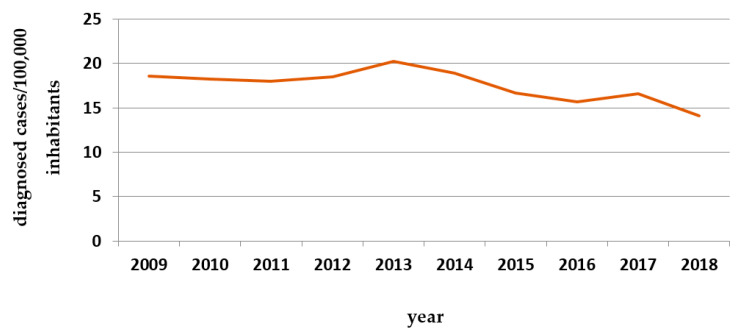
Number of cases diagnosed in Portugal with “diabetic foot” per 100,000 inhabitants (hospital admissions) since 2009 to 2018. Source: INFARMED [13].

**Figure 3:**
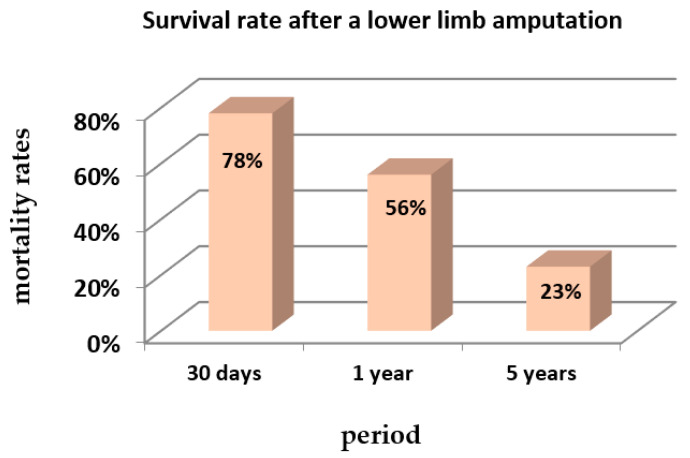
Mortality rates after a first lower limb amputation (%) in a period of 5 years—retrospective cohort study. Source: Fortington et al., 2013 [14].

The following figures contain data on the incidence of diabetic foot (Table 1 and Figure 2) and mean survival after lower limb amputation (Figure 3), two of the most common complications in diabetic patients.

**Table 1:** Prevalence of diabetic foot ulcers in different countries in the population of patients with DM2 followed at the hospital level and in public health study centers. Source: Pengzi Zhang et al., 2017 [12].

Country	No. of Studies	Prevalence	Country	No. of Studies	Prevalence
Belgium	1	16.6%	South Africa	2	5.8%
Canada	1	14.8%	France	1	5.6%
USA	3	13.0%	Greece	1	4.8%
Trinidad	1	12.2%	Jordan	2	4.2%
India	2	11.6%	China	10	4.1%
Norway	1	10.4%	Uganda	1	4.0%
Cameroon	3	9.9%	Ireland	1	3.9%
Italy	1	9.7%	Turkey	1	3.1%
Thailand	2	8.8%	Spain	5	3.0%
Denmark	1	7.8%	Germany	2	2.8%
Pakistan	4	7.4%	Saudi Arabia	1	2.3%
Tanzania	2	7.3%	Japan	1	2.0%
Pacific island countries	1	6.8%	Netherlands	2	1.8%
United Kingdom	4	6.3%	Korea	2	1.7%
Egypt	2	6%	Poland	1	1.7%
Bahrain	1	5.9%	Australia	2	1.5%

The burden of suffering due to diabetes is increasing despite significant investment in clinical care and pharmaceutical research. Notably, Western Europe has a rate of increase greater than that of global and Asian averages. Even with the high levels of clinical and public health expenditure, this region is losing the battle against diabetes. One explanation might be non-modifiable risk factors, such as age and family history [15]. In fact, global aging, economic growth, rapid and trending urbanization, as well as nutritional transitions (to a highly processed, high-calorie diet) associated with a sedentary lifestyle, exacerbate this trend [16].

**Figure 4:**
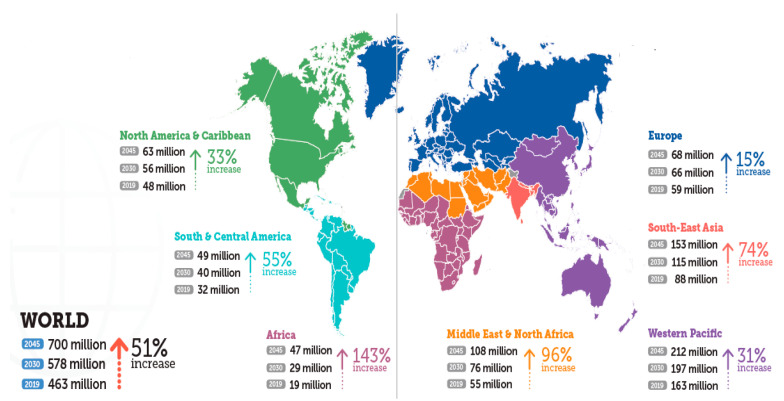
Number of people with diabetes worldwide and per IDF region in 2019, 2030 and 2045 (age 20–79 years). Source: IDF Diabetes Atlas editions [8].

According IDF, and Figure 4 illustrates this reality, developed countries endure the highest burdens of human suffering due to diabetes and some findings support the correlation between diabetes and economic development [17].

These findings have direct implications for health systems planning and resource allocation. Clearly, hospital-based management and subspecialist care are not sustainable strategies. Resource allocation in healthcare budgets for the prevention of diabetes, promoting health behaviors that are consistent with more effective disease prevention, needs to be comparable to expenditures on treatment [18].

The consequences associated with erratic behavior, both in terms of adherence to therapy and in terms of lifestyle, preventing adequate glycemic control can trigger potential complications, including cardiovascular disease, peripheral neuropathy, kidney disease, retinopathy, skin disorders, hearing changes, sleep apnea or neurological disorders associated with dementia, which can be extremely disabling and compromise the life expectancy of patients [19,20].

In order to ensure an acceptable level of quality of life in the face of the clinical condition of diabetes, it is essential that patients adopt appropriate behaviors in terms of adherence to therapy and that they are complemented with a healthy lifestyle, with health professionals taking on a fundamental role in terms of education and guidance in this regard. In this context, the behavioral sciences meet this premise, aiming to assess how human actions can contribute to a greater or lesser effectiveness of the pharmacological and non-pharmacological measures instituted and, therefore, how they influence the quality of the disease’s prognosis [20].

There is some scarcity of studies addressing the topic of diabetes mellitus in an interdisciplinary way, with most published studies addressing disease behaviors as being essentially clinical and pharmacological in nature, without reflection on the role that behavioral sciences can play in disease management. To address this gap, our aim with this narrative review is to address the potentially modifiable factors that can influence the adoption of appropriate behaviors by patients with DM2, in order to enable an adequate treatment and control of the disease, emphasizing the role of behavioral sciences. It is a global approach that, in addition to aspects directly related to lifestyle and adherence to pharmacological therapy, intends to provide a historical and statistical context of the disease, complemented with a brief summary of its pathophysiology, of the pharmacological therapies currently available as well as of elementary concepts for the understanding and applicability of behavioral sciences. Since health professionals need to carry out a constant review and update of knowledge in order to optimize their professional practice, the growing knowledge about diseases, the continuous emergence of new therapeutic formulations, as well as the definition of strategies each time more rigorous in terms of influencing behaviors, highlight the importance of carrying out review studies in these areas that are capable of offering a holistic view of all the factors, bringing together those which may impact on the control and treatment of chronic diseases.

## 2. Materials and Methods

**Figure 5:**
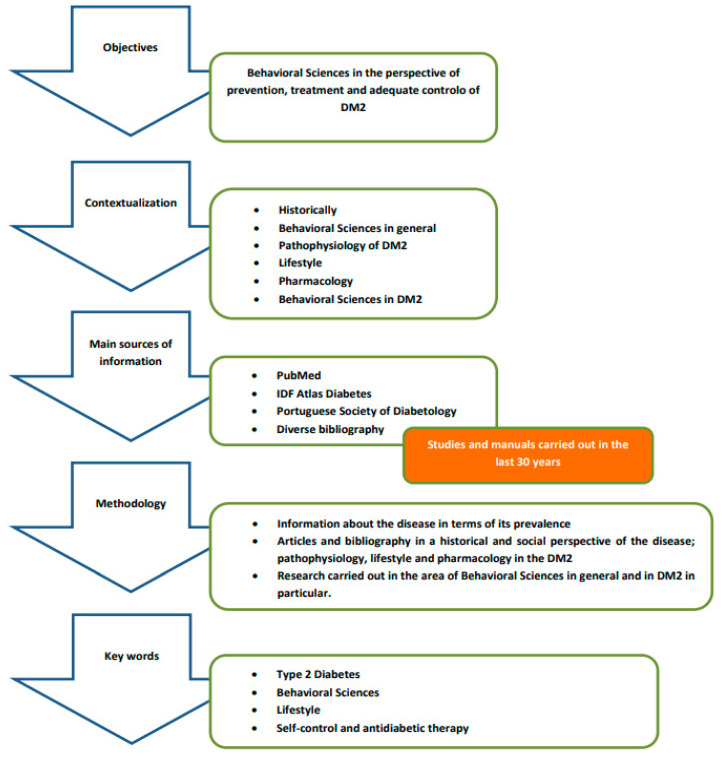
Research methodology and information synthesis.

A narrative review was developed to synthesize the findings published in the literature, retrieved through a search of databases, manuals, previously published scientific articles and official texts, following the Scale for the Assessment of Narrative Review Articles (SANRA) methodology as schematized in Figure 5 [21].

- The guiding principle used in this review, which, in the end, defines the central issue of this work, is the importance of behavioral sciences in the perspective of prevention, treatment and adequate control of DM2, so the adoption of assertive behaviors assumes an extreme preponderance in achieving positive results, with health professionals having a decisive role in defining effective strategies to ensure that patients can assume a commitment, at a behavioral level, leading to the success of pharmacological and non-pharmacological treatments instituted.
- This narrative review intends to contextualize the disease from a historical point of view; summarize the essence of behavioral sciences and what is its framework; discuss what kind of approach should be taken both in terms of lifestyle and pharmacologically, given the pathophysiology of DM2, reviewing the therapeutic options currently available and its main characteristics that can influence the behavior of patients; and address the potentially modifiable aspects that are decisive in influencing the behavior of patients with DM2.
- The main sources of information were databases such as PubMed, IDF Diabetes Atlas, the Portuguese Society of Diabetology, as well as manuals in the field of Pharmacology/Pharmacotherapy, focusing generically on search terms such as behavioral sciences, history, pathophysiology, lifestyle and complications/comorbidities in diabetes, and specifically guidelines for the treatment of DM2 and behavioral sciences in diabetes.
- The key statements are supported by references to studies carried out in the past 30 years in the field of behavioral sciences as well as on the history, incidence, pathophysiology and drug therapy of DM2. We used a three-stage approach to review the literature:
  1. The first stage consisted of researching statistical information about the disease in terms of its prevalence.
  2. The second stage refers to the research of articles and bibliography in a historical and social perspective of the disease; pathophysiology, lifestyle and pharmacology in the DM2.
  3. The third stage reports on research carried out in the area of behavioral sciences in general and in DM2 in particular.
- The initial keywords were organized into the following conceptual categories: type 2 diabetes, behavioral sciences, lifestyle, self-control and antidiabetic therapy. Search terms were developed and customized for each database.
- The data and information gathered are presented establishing a logical sequence that aims to demonstrate how it is possible to identify and act to ensure adequate control of DM2 from the behavioral perspective of patients with the disease.

## 3. Main Findings

### 3.1. Historical and Social Context of the Disease and Behaviors

The first known mention of diabetes symptoms was in 1552 BC, when Hesy-Ra, an Egyptian physician, documented frequent urination as a symptom of a mysterious illness that also caused weight loss. Also at this time, ancient healers recorded that ants seemed to be attracted to the urine of people who had this disease [22].

In AD 150, the Greek physician Arateus described what we now call diabetes as “the melting of flesh and limbs in urine” [23].

Centuries later, people known as “water testers” diagnosed diabetes by sampling the urine of people suspected of suffering from the disease. If the urine tasted sweet, diabetes would be diagnosed [24].

During the Franco-Prussian War in the early 1870s, the French physician Apollinaire Bouchardat observed that the symptoms of his diabetic patients improved due to food rationing caused by the war, and from then on, he developed individualized diets for the treatment of diabetes. This logic led to very common diets in the early 1900s, which included the “oat cure”, the “potato therapy” and the “hunger diet” [25].

In 1916, scientist Elliott Joslin established himself as one of the world’s leading diabetes experts by creating the book *The Treatment of Diabetes Mellitus*, which reported that a fasting diet combined with regular exercise could significantly reduce the risk of death in patients with diabetes [26].

The association of insulin shortage with the disease in the first half of the 20th century radically changed the approach to the disease, marking the beginning of a therapeutic revolution in this area.

Today, healthcare professionals still use some of these principles when modulating their patients’ behavior towards lifestyle changes that allow for adequate disease control [24,27,28].

In the first half of the 20th century, public health was mainly dominated by a biomedical perspective, and only after the Second World War was it also framed within a social science perspective. From that period on, there was a theoretical development in economics, political science, sociology and anthropology, re-dimensioning the concept of public health, which previously focused mainly on the individual, now coming to understand the entire social structure [2,6]. It is also from this moment that many individual behaviors were not only recognized as risk factors for health problems, but also began to be inserted in a broader social context. Public health policy, based on social sciences, was the key to the development of strategies capable of promoting appropriate practices in the promotion of healthy lifestyles, with a clear impact on the prevention and control of several chronic diseases such as DM2 [6,29].

### 3.2. The Essence of Behavioral Sciences

Behavioral sciences generally correspond to a branch of science (such as psychology, sociology or anthropology) directly applicable to human action, with the aim of generalizing about human behavior in society.

A patient’s psychological aspects such as personal values, beliefs, cognitive function and emotion form the basis of behaviors in human health, which, in turn, influence self-management, self-efficacy, quality of life, control and clinical outcomes in patients with chronic diseases [30].

Whitley and Kite (2013) argue that the behavioral sciences are based on three fundamental and closely related principles: research that generates knowledge, theory that organizes knowledge and application that puts knowledge to use [2].

The main objective of science in behavior change is to improve our understanding of the mechanisms of action of the interventions that are intended to be implemented [31].

Behavior theories identify potential “determinants” of behavior, that is, factors that can influence the behavior under analysis. These determinants are the pathways through which behavior change techniques are applied in order to achieve the intended results [32,33].

Currently, behavior change intervention mechanisms are typically studied using mediation analyses, where the impact of X (an intervention) on Y (a behavioral outcome) is adapted to be subject to a third variable M (for example, a theoretical determinant of the behavioral outcome targeted by the intervention). In the presence of classical mediation, the X-Y path would be reduced to almost zero when variable M is added to the model. In the case of behavior change interventions, one could conclude that intervention (X) changed behavior (Y) because it changed important theoretical determinants of behavior (M) [34].

In an analysis of behavioral mechanisms, its accuracy tends to be simpler if certain requirements are met, such as [34]:

- The number of variables involved is small and the dynamics can be significantly evaluated in just a few points of time;
- The change process is the same for all individuals, e.g., following the same sequence;
- The dynamics between variables is linear, additive and does not change over time; and
- Included variables are not diluted in context or omitted.

However, human behavior is complex, and although theories have been formulated as close as possible to linear methods of analysis, this approach can mask important characteristics of behavior change. Linear models are inadequate in most studies related to behavioral sciences, because on the one hand, there are many non-linear interactions in the time scale [35,36,37,38], and on the other hand, traditional statistical analyses start from the simplification that everything is regardless of what surrounds it [39,40].

To face this limitation, complex adaptive systems (CAS) have emerged, which represent a more useful analytical lever for behaviors [41]. A CAS involves several levels of interaction between heterogeneous agents; not all intervention components are created equally, thus giving it a non-linear nature. It is a system that is composed of heterogeneous actors and the behavior of each responds to the actions of other people within the system (it is adaptive) [42]. The agents within a CAS are interconnected in such a way that each action of an individual affects the context of the other agents—with indirect implications for all subsequent behavior [43]. In this case, the control of behavior is distributed rather than hierarchical. It is the ability to respond to change between agents, acting locally within the CAS, that gives such systems their dynamic, responsive and productive nature [44]. Due to the dynamic and changing nature of social contexts, actors within a complex system are constantly adapting to changes at the local level. If social behavior evolves in this way, a CAS does not reach stagnation—instead, it expresses a double movement between stability and instability; regularity and irregularity [45].

Understanding the mechanisms that can influence the behavior of patients in order to promote optimal adherence to therapeutic plans in the treatment of chronic diseases requires a comprehensive vision that understands the dynamism of the environment in which they live, as well as their individual characteristics that may affect whole the process.

### 3.3. Pathophysiology of Type 2 Diabetes

Obesity and sedentary lifestyle are states of insulin resistance that, when associated with genetic factors, adversely affect the functioning of pancreatic β cells, which, in a compensatory way, increase insulin secretion. An excessive intake of carbohydrates, associated with a malfunction in the feedback circuits between the action of insulin and its secretion, results in abnormally high levels of glucose in the blood [3,19,46,47]. The maintenance of this compensatory state in insulin secretion leads, over time, to the appearance in an initial phase of high levels of postprandial glucose, in a second phase of fasting glucose, ultimately culminating in the onset of diabetes mellitus of the type 2 [4,19]. If the dysfunction is present in the β-cells, insulin secretion is reduced, limiting the body’s ability to maintain physiological glucose levels. On the other hand, insulin resistance contributes to an increase in the hepatic production of glucose and a decrease in its uptake by muscle, liver and adipose tissue [4,19,48]. β-cell dysfunction is generally more severe than insulin resistance, but when both are present, hyperglycemia is amplified, leading to DM2 progression [49,50,51]. This dysfunction has traditionally been associated with β-cell death; however, recent evidence suggests that it may be associated with a more complex network of interactions between the environment and the different molecular pathways involved in cell biology [52]. Hyperglycemia results from the combination of different pathophysiological anomalies ranging from resistance to insulin action (both liver and muscle), by its inadequate secretion, by excessive or inappropriate glucagon secretion, by reduced incretin effect, by increased lipolysis, due to increased renal glucose reabsorption and dysfunction of brain neurotransmitters [5,51,53,54]. In the face of an excessive nutritional status, similar to that found in obesity, hyperglycemia and hyperlipidemia are often present, promoting insulin resistance and chronic inflammation. Under these circumstances, β cells, due to differences in terms of genetic susceptibility, are subject to toxic pressures, inflammation and metabolic/oxidative and amyloid stress, with the potential to ultimately lead to a loss of their integrity [47,50,52,53].

DM2 patients often present, in terms of symptoms, lethargy, polyuria, nocturia and polydipsia [5,51].

### 3.4. Healthy Lifestyle

Therapeutic success in DM2 depends on the effectiveness of the pharmacological treatments instituted, which must be complemented with a healthy lifestyle, so it is essential that adequate behavioral practices are ensured both in terms of a balanced/adequate diet as well as by the need for regular physical exercise, especially in obese patients who are at an increased risk of developing the disease [55,56,57,58].

From a dietary point of view, compliance with certain recommendations allows not only preventing the onset of the disease, but also delaying/preventing its progression. A decrease in the intake of added sugars and processed foods; an increase in the intake of fiber, fruits and vegetables; a reduction in the intake of processed meat and red meat; and the intake of healthier fats are key aspects for a more balanced diet, capable of contributing to a significant improvement in the clinical prognosis of the disease [59,60].

Physical exercise is also extremely important in the prevention and control of DM2, and the incidence of the disease is inversely proportional to the participation in physical activities, and this relationship was demonstrated by the systematic review by Warburton et al. (2010) when they analyzed several cohort studies in this area [58,59,60,61,62].

Weight loss is important for the prevention of DM2, and studies of intensive lifestyle intervention have shown that there was a 16% reduction in the risk of diabetes per kilogram of weight lost [60,62].

The patient must be made aware of the importance of strict adherence to therapy as well as the adoption of a healthy lifestyle so that the benefit of pharmacological therapy is maximized, being essential the differentiating intervention of health professionals in this regard, both by the need to promote good practices as well as to ensure ever more rigorous information channels are ensured, especially in an era where very unreliable sources of information proliferate [63,64].

According to the World Health Organization, “without a system that addresses the determinants of adherence, advances in biomedical technology will fail to realize their potential to reduce the burden of chronic disease” [65].

According to the clinical guidelines for the treatment of diabetes, there are three fundamental principles that must be adhered to in order to ensure adequate control of the disease [66]:

1. Lifestyle changes with the adoption of adequate eating habits and physical exercise throughout the course of the disease;
2. Individualization of therapy and patient-centeredness; and
3. Therapeutic Education (TE) or DSMES (Diabetes Self-Management Education and Support), which is essential in the care provided to people with type 2 diabetes.

Since patient-centeredness is one of the fundamental points in the treatment and control of DM2, behavioral sciences play a fundamental role at this level, especially in terms of their inclusion in informed and shared decision-making. Some studies estimate that one in three patients do not comply with the established therapeutic regimen, with higher non-compliance rates among racial/ethnic minorities and in people with low socioeconomic status [67,68]. The impact of non-adherence to the instituted therapy leads to an increase in morbidity and mortality rates, in addition to having an impact on costs at the level of healthcare systems [69,70,71].

There are several factors that contribute to non-adherence to therapy, such as side effects of medication, disbelief in the treatment, lack of motivation, deterioration of the relationship between the health professional and the patient, difficulties in accessing treatment, impaired cognitive function and inability to adopt healthy lifestyle habits [67,68].

It is essential that communicational strategies are adopted that influence behaviors, especially by selecting theories of behavioral influence, which provide patients with DM2 with assertive behavior and that allow for the proper treatment and control of the disease [70,71,72]. An adequate theory should demonstrate effectiveness in predicting behavior, focused on modifiable targets, able to provide a sufficient description of how targets explain or mediate effects on behavior (specific pathways), and include measures that properly operationalize the intended targets to drive behavior change, following the information-motivation and behavioral skills (MBS) model to these premises [71,72,73,74,75,76].

Briefly, the MBS model defends that an informed patient, motivated and available to act, has the skills and confidence to make more assertive decisions that will allow greater success in the desired results. Patient/disease characteristics can be classified as being generally non-modifiable or potentially modifiable [77,78]. Poor adherence to therapy is associated with factors that may not be directly related to the patient (such as lack of integrated care at the level of health systems and clinical inertia among health professionals), demographic factors (young age, low level of poor education and financial situation), the patient’s beliefs about the therapy (perceived treatment ineffectiveness) and the implications for the patient that are directly related to the therapeutic regimen (treatment complexity, direct costs and hypoglycemia) [77,78,79]. Aspects such as the risks potentially associated with hypoglycemia and other adverse drug effects, the duration of diabetes (whether the diagnosis is recent or long-term), the life expectancy, concomitant pathologies and established vascular complications are generally not modifiable [78]. The patient’s attitude and motivation, as well as their capacity for self-care, together with the resources and support systems at their disposal, are potentially modifiable aspects that can decisively influence the disease’s evolutionary state [77,78,79,80].

Strategies aimed at low adherence should focus not only on reducing the impact of therapy in terms of adverse events, but also on addressing and correcting patients’ negative beliefs regarding medication. In order to overcome these obstacles, it is essential to use methodologies based on behavioral innovations, as well as new methods and modes of drug administration [77,78]. The specific barriers to adherence to therapy in type 2 diabetes, especially those that are potentially modifiable, should be identified more rigorously and should be seen as a priority target for action in terms of behavioral influence.

It is important, first of all, to know some concepts that are often used interchangeably in the description of behavior in patients’ compliance with a therapeutic regimen, namely adherence (to what extent a patient’s behavior—taking medication, following a diet and/or making lifestyle changes—corresponds to the recommendations agreed with a health professional), agreement (joint agreement between the prescriber and the patient regarding therapeutic decisions, including the use of prescribed drugs in a certain way), compliance (to what extent the patient’s behavior corresponds to the prescriber’s recommendations) and persistence (the length of the process use of a drug) [81]. However, the use of the term “compliance” has fallen into disuse, as it suggests a lack of patient involvement [81].

### 3.5. Pharmacological Treatment

Treatment includes the definition of a glycemic target with a view to its normalization (with most patients having an HbA1c < 6.5%, which should be evaluated every 3 months), educational measures, evaluation of micro and macrovascular complications, monitoring and control of cardiovascular risk factors and, in the long term, avoiding drugs that may exacerbate inappropriate insulin levels or influence lipid metabolism [54,66,82].

The selection of an appropriate “p-treatment” should follow defined guidelines (consensus report by the American Diabetes Association—ADA—and the European Association for the Study of Diabetes—EASD), considering the intended therapeutic objectives and inter-individual and intra-individual variability (age, life expectancy and concomitant comorbidities) [66,82]. It is essential that certain risk groups be considered and classified, in which the treatment of hyperglycemia requires the use of specific references, such as in cases of cardiovascular patients or patients with chronic kidney disease, in the most debilitated elderly, in patients in whom hypoglycemia occurrence is potentially more serious and even in the obese. Given the specificities inherent to the treatment of hyperglycemia in these populations, these recommendations include specific references for these situations [66,83].

Over the past few decades, we have witnessed the market introduction of different therapeutic formulations, varying in terms of mechanisms of action, routes of administration and even the emergence of fixed-dose combinations, which have, on the one hand, allowed the therapeutic objectives to be achieved and pre-defined clinical stability and, on the other hand, facilitate/optimize adherence to therapy, bypassing behavioral barriers that may compromise the success of established therapeutic regimens [48,84].

The available pharmacological treatments focus on increasing the availability of insulin (either through the direct administration of insulin or the administration of drugs that promote its secretion), increasing insulin sensitivity and reducing or preventing the absorption of glucose at the level of the gastrointestinal tract, by increasing urinary glucose excretion or by a combination of several approaches (Table 2).

The association of drugs with different mechanisms of action to benefit from the synergistic effect should be privileged. The combination of three oral antidiabetics may possibly be considered; however, if necessary, the early initiation of insulin should be considered in patients who have difficulty in achieving the defined goal in therapeutic terms [51,54].

### 3.6. Potentially Modifiable Factors in Which Efforts Should Be Made to Influence the Behavior of Patients with DM2

#### 3.6.1. The Effectiveness of the Treatment According to the Patient’s Perception

Patients are more likely to adhere to treatment regimens when they have some tangible sense that the prescribed medication contributes to relatively immediate positive outcomes.

In DM2, most patients are asymptomatic, and the pathology can only be diagnosed after blood analysis. However, in more advanced/severe stages of the disease, symptoms such as lethargy, polyuria, nocturia and polydipsia are frequent, mainly in overweight patients [51].

The benefits resulting from the fulfillment of a certain therapeutic plan that makes it possible to correct/attenuate the symptoms undoubtedly contribute to a more effective adherence by the patients and better self-control [87,88,89].

#### 3.6.2. The Incidence of Hypoglycemia

The fear of a hypoglycemic crisis is generally considered one of the main barriers to achieving adequate DM2 control [89,90,91,92,93].

A cross-sectional study of DM2 patients treated with metformin and a sulfonylurea demonstrated that patients who reported moderate or severe symptoms of hypoglycemia had conditioned medication adherence compared to those without mild hypoglycemia or without hypoglycemia [92,93].

According to Polonsky and Henry study [76], 56% of patients with type 2 diabetes had experienced hypoglycemia and had higher HbA1c levels compared to the rest.

Strategies must be adopted by health professionals who work at the level of prevention of the risk of hypoglycemia, focused on patient education and also favoring the use of new therapeutic agents that are associated with a lower incidence of hypoglycemic events [94].

#### 3.6.3. Complexity and Convenience of Treatment

Adherence to therapy becomes more challenging when the treatment itself is seen as more complex, costly or painful [95,96]. Treatment persistence is a key element associated with the effectiveness of pharmacological therapies in DM2 patients.

Antidiabetic drugs act through different mechanisms of action; therefore, the definition of the therapeutic regimen must consider the characteristics of the patients, the mode of administration (oral or injectable), the intended glycemic effect and the potential risks (hypoglycemia, weight gain, cardiovascular safety profile and side effects) [96,97].

In a comprehensive review including 76 studies, Claxton et al. (2010) concluded that, for polymedicated patients (with several prescribed drugs and with multiple daily doses), there is a decreasing adherence to the treatment plan [98].

Studies carried out on the relationship between the onset of depressive symptoms and glycemic control in DM2 have shown that patients undergoing insulin therapy are more prone to depression than those undergoing treatment only with oral formulations, this pattern being related to the greater complexity of the regimen and the need for multiple daily injections [99,100,101].

The convenience or complexity of drug delivery devices can also influence adherence. Some studies have shown that with the use of an insulin pen instead of the use of a bottle and syringe in patients with DM2, adherence, glycemic control and lower incidence rates of hypoglycemia were obtained in the groups of patients treated with insulin pens instead of syringes [77,90]. In the case of oral formulations, patients who were previously treated with only one drug and who need additional therapy show significantly greater adherence when the regimen comprises fixed-dose combined therapy compared to combined therapy using each of the agents separately; similarly, patients who receive combination therapy and who switch to fixed-dose combination regimens also adhere to the treatment plan more strictly after the switch [102]. The most recent oral formulations (i-DPP4, i-SGLT−2), with greater therapeutic efficacy, greater dosage convenience (possibility of a single daily dose) and better tolerability by patients, are associated with higher rates of persistence in the treatment of DM2 than with metformin and sulfonylureas, and the route of administration also influences this parameter (GLP−1 agonists, although being of the new generation, are administered subcutaneously and, given the i-DPP4 and SGLT−2, they have less persistence in the treatment) [93,103].

One of the aspects that is closely associated with the lesser efficacy of oral antidiabetic therapy is related to forgetting to take the medications, leading to fluctuations in glycemic levels, being important at this level the reinforcement of the educational component by health professionals [104].

#### 3.6.4. Costs of Treatment

Health costs must be analyzed in two ways: direct costs, which refer to the amount paid by patients when purchasing therapy, and which have been consistently associated with non-adherence in all conditions of treatment of chronic diseases, especially at higher purchase values; and indirect costs, when a disease that is poorly controlled, resulting from inadequate patient adherence to treatment, is closely associated with an increased probability of needing hospital admission and more costly therapeutic approaches [105,106,107].

**Figure 6:**
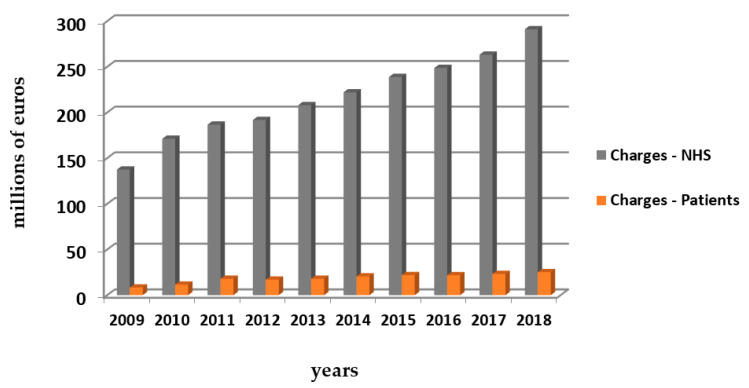
Outpatient sales of insulin and non-insulin drugs within the NHS in Continental Portugal—in value (millions of euros) since 2009 to 2018. Source: Raposo J., 2020 [13].

**Figure 7:**
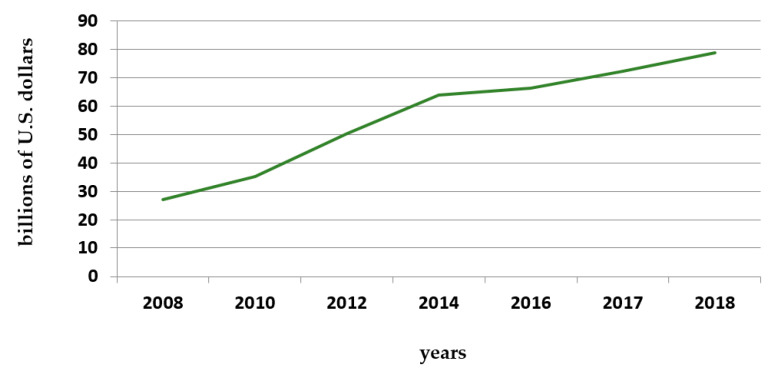
Global pharmaceutical revenue of anti-diabetic products in billions of U.S. dollars from 2008 to 2018. Source: Mikulic 2020—Statistics & Facts [105].

Diabetes caused at least USD 760 billion in health expenditures in 2019, comprising 10% of total spending on adults [8]. The following graphs (Figure 6 and Figure 7) provide an idea about the costs of antidiabetic medication in Portugal and worldwide.

When defining government strategies in the health area, the correlation between the increase in non-adherence and the higher prevalence of the disease must be considered, so that the available resources are managed more efficiently, assuming patient education, with strategies of behavioral intervention, a fundamental role in this issue [107,108].

#### 3.6.5. Beliefs Regarding Medication

Health behaviors can be affected both by patients’ literacy and their self-management capacity. Cognitive and social skills are directly related to the motivation of patients, influencing it, which is decisive in the ability to assimilate knowledge, which, in turn, contributes to a better understanding of the intended therapeutic goals [109]. Many patients have markedly negative or highly skeptical beliefs about therapy, often fearing that the long-term risks will outweigh any likely benefits [110,111].

In patients with DM2, concerns about poor adherence to the defined therapeutic regimen demand from health professionals an objective approach in relation to the clear demonstration of benefits for the patient through correct adherence, as well as the identification of barriers/socio-psychological factors that may negatively influence strict compliance with the established regime, allowing assertive action in this regard [109,110,111].

#### 3.6.6. Trust in Health Professionals

Optimum adherence to treatment with hypoglycemic drugs is closely related to a trusting relationship between health professionals and patients [112,113]. The consonance/complicity between patient and physician (a patient’s feeling that their needs and concerns, during medical consultations, were heard and attended to) predicts the quality of adherence to pharmacological treatment in the long term, contributing to the prevention of complications and, when established, to a greater effectiveness in their control [114,115].

Pharmacists also assume a differentiating role in this domain, both in a hospital context and in a community environment, interpreting the medical prescription by the identification of any non-compliance with the prescribed medication (if it appears to be unsafe to use with a patient’s other medications, if the dose or duration is inappropriate or if the cost is overly burdensome). They have the ability to recommend appropriate alternatives by reaching out to the prescriber, as well as when performing therapeutic reconciliation, ensuring the efficacy and safety of the defined therapeutic regimens. They can play an integral role in diabetes control, either by educating patients about lifestyle changes and medications, or even managing the general state of the disease [116,117].

Nurses are also extremely important in this dynamic. Its intervention includes not only instruction in the administration of medications and treatments (for example, in the “diabetic foot”), but also psychological support so that patients can be better able to face the daily challenges of a chronic condition [118].

Education is the cornerstone of health. The International Diabetes Federation (IDF) advocates the sharing of information and continuously improved practices in the field of diabetes in order to equip healthcare professionals with the best understanding and skills to be able to provide optimal care and support to their patients. 

## 4. Discussion

Focusing on potentially modifiable aspects in the approach to the treatment and control of diabetes, it is possible to see that the behavioral, motivational and self-care components of the patient, combined with their cognitive capacity and the wealth of information they have at their disposal, undoubtedly contribute to their success of the pharmacological and non-pharmacological therapeutics instituted [119,120,121]. In addition to aspects directly related to drug therapy (the right drug, in the right dose, at the right time), it is essential to ensure a healthy lifestyle (balanced diet with sugar restriction, adequate exercise, sleep hygiene, avoid the consumption of alcohol, smoking or consumption of illegal substances), so that the intended therapeutic effect is maximized, that acute exacerbations of the disease are prevented and, consequently, the incidence of morbidity and mortality is reduced. Although several studies have shown that adherence to treatment is associated with better control [122,123,124], almost 60% of patients with diabetes are unable to reach their glycemic goals, so it is essential to understand the factors associated with non-adherence, so that patient-focused strategies can be developed, enabling effective intervention by health professionals and thus reducing complications associated with uncontrolled diabetes and, on the other hand, the potential impact in terms of health expenditure.

Scientific advances in the development of new treatments and improvements in drug delivery systems, together with innovative technologies, can and should be used to help patients overcome some of the challenges associated with controlling the disease.

## 5. Conclusions

Diabetes is a pathology that has a strong psychological burden for its patients, both because of the need for thorough control of glycemic levels, implying strict adherence to the instituted therapeutic regimen, and because of the indispensability of adopting healthy lifestyle habits, due the side effects of disease and the therapy itself, as well as fears arising from acute clinical conditions (hypoglycemia).

More research is needed to promote a more effective discussion on the importance of investing in the social and behavioral component of the chronically ill, as also concluded in several recent studies. It is important to understand how patients live with chronic diseases such as DM2, the impact it has on their lives and how it can be minimized through the influence of behaviors, especially by the intervention of health professionals, combining this aspect with knowledge about the pathophysiology of DM2 and about the pharmacological characteristics of available therapies. 

Health systems must evolve towards increasing efficiency of the services provided, guaranteeing all patients with the appropriate clinical, pharmaceutical and social follow-up to each need, as well as minimizing potential complications that could compromise the quality and life expectancy of patients, with natural implications in terms of economic expenditure.

## Figures and Tables

**Table 2:** General characteristics of anti-diabetic therapy [4,5,51,54,83,84,85,86].

Drug	Mechanism of Action	Risk of Hypoglycemia	Weight	Secondary Effects
Metformin (*)	↓ hepatic glucose synthesis	Not associated	↓	GI changes (diarrhea and vomiting) and vitamin B12 deficiency
α-Glucosidase inhibitors (**)	Prevent the breakdown of complex carbohydrates in the small intestine, delaying their absorption	Not associated	=	Diarrhea, flatulence or abdominal discomfort
Sodium-glucose cotransporter inhibitors (SGLT2)	↑ elimination of glucose in the urine and block its renal absorption	Not associated	↓	↑ risk of genitourinary infections, hypovolemia with hypotension, increased LDL cholesterol and may even lead to a transient increase in creatinine
Glucagon-like peptide−1 agonists (GLP−1 agonists)	↑ insulin secretion, by decreasing glucagon secretion, delaying gastric emptying, also promoting the feeling of satiety	Low	↓	Nausea, diarrhea, vomiting, and headache
Dipeptidyl peptidase 4 inhibitors (iDPP4)	Inhibit the degradation of incretins which promote↑ secretion of insulin and the ↓ of glucagon secretion	Not associated	=	Well tolerated
Thiazolidinediones	↑ peripheral insulin sensitivity in liver, fat and skeletal muscle cells	Not associated	↑ but ↓ visceral obesity	↑ risk of fluid retention (edema), congestive heart failure and an increased risk of bone fractures
Sulfonylureas and Glinides (***)	Secretagogues (↑ insulin secretion)	Increased risk	↑	Well tolerated
Insulins (****)	Activates insulin receptors	High	↑	Possibility of local allergic reactions

* Considered the first line in the treatment of type 2 diabetes if there are no contraindications to its use [66]. ** Useful in reducing postprandial glycemia and promoting a modest reduction in HbA1c [81]. *** Glinides are useful for patients with erratic behavior (irregular meal times or skipping meals). They have a similar risk for inducing weight gain as sulfonylureas do, but possibly carry less risk for hypoglycemia [54]. **** Available insulins are classified according to their duration of action, as slow or basal (intermediate and long acting) and short/fast acting, and there are also biphasic formulations (pre-mixes) [54,85,86].
